# Multisystemic Manifestations of Hyaline Fibromatosis Syndrome: Implications for Diagnosis and Management

**DOI:** 10.7759/cureus.47250

**Published:** 2023-10-18

**Authors:** Noor Albusta, Hasan M Isa, Halima E Al-Jowder

**Affiliations:** 1 Internal Medicine, Salmaniya Medical Complex, Manama, BHR; 2 Pediatrics, Arabian Gulf University, Manama, BHR; 3 Pediatrics, Salmaniya Medical Complex, Manama, BHR

**Keywords:** protein-losing enteropathy, joint contractures, skin nodules, faltering growth, hyaline fibromatosis syndrome

## Abstract

Hyaline fibromatosis syndrome (HFS) is a rare autosomal recessive disorder characterized by the deposition of hyaline material in the skin, soft tissues, and bones. In this report, we discuss a case of a six-month-old male with HFS who presented with faltering growth, chronic diarrhea, multiple joint contractures, joint stiffness, hyperpigmented skin over bony prominences, gingival hypertrophy, patent foramen ovale, and symmetric periventricular hyperintensities on brain MRI. The diagnosis of HFS was confirmed by skin biopsy and genetic testing, which identified a homozygous mutation in the anthrax toxin receptor 2 (ANTXR2) gene. The patient was managed symptomatically with nutritional support, physiotherapy, analgesics, and regular dental care. He also received intralesional corticosteroid therapy, which significantly decreased the size of the skin nodules. His hyperpigmented skin and gingival hypertrophy remained stable, and the patent foramen ovale was managed conservatively. This case report highlights the importance of early diagnosis and management of HFS and the benefits of involving a multidisciplinary team to improve the quality of life of affected individuals.

## Introduction

Hyaline fibromatosis syndrome (HFS) is a rare genetic disorder; it was first described by Murray et al. in 1873 [1]. HFS forms part of a group of disorders known as "hyalinosis," which is characterized by the deposition of hyaline material in various tissues and organs [2]. It affects both males and females equally and is not predominantly associated with any particular ethnic or racial group [3]. It is inherited in an autosomal recessive manner and is believed to be caused by mutations in the anthrax toxin receptor 2 (ANTXR2) gene. The ANTXR2 gene, also known as the capillary morphogenesis gene 2 (CMG2), is located in chromosome 4q21.21 and encodes a transmembrane protein called anthrax toxin receptor 2. The protein is involved in the transportation of collagen fibers and other extracellular matrix components. The role of ANTXR2 in the pathogenesis of HFS is not fully understood, but it is thought that mutations in this gene disrupt the transportation of extracellular matrix components, leading to the deposition of hyaline material in various tissues and organs [4].

The clinical manifestations of HFS vary widely and can affect various parts of the body. The most common symptoms of HFS are skin lesions, joint contractures, and bone deformities. Skin lesions in HFS usually constitute firm nodules that appear on the face, scalp, trunk, and extremities. The skin lesions can also ulcerate and become infected, leading to scarring and disfigurement. Joint contractures in HFS usually involve the elbows, knees, and ankles, which can lead to pain, stiffness, and limited mobility. Bone deformities in HFS can affect various bones in the body, resulting in malformation and abnormal growth [3,4].

The diagnosis of HFS is based on clinical presentation and confirmed by genetic testing [1,2,4]. We report a case of a six-month-old Bahraini male who presented with faltering growth, joint contractures, and skin nodules during early infancy and was diagnosed with infantile HFS following the identification of the homozygous mutation in the ANTXR2 gene. This is the second case of HFS reported in Bahrain and the first to involve prominent gastrointestinal manifestations in this region [5].

## Case presentation

**Figure 1:**
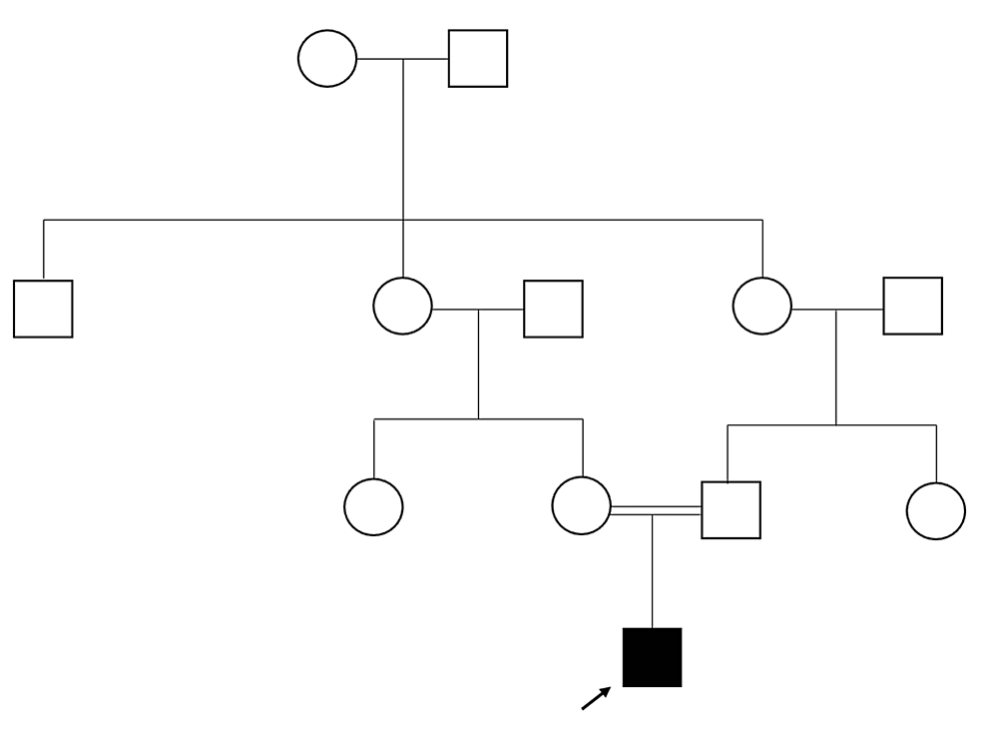
Pedigree of the patient (arrow) The patient’s parents were consanguineous and there was no family history of any metabolic disorder

A six-month-old Bahraini male was brought to the pediatric clinic with a history of faltering growth, chronic diarrhea, multiple skin lesions, and joint stiffness since early infancy. He was the first child of healthy consanguineous parents, with a negative family history of similar conditions. Figure 1 shows the pedigree of the patient.

**Figure 2:**
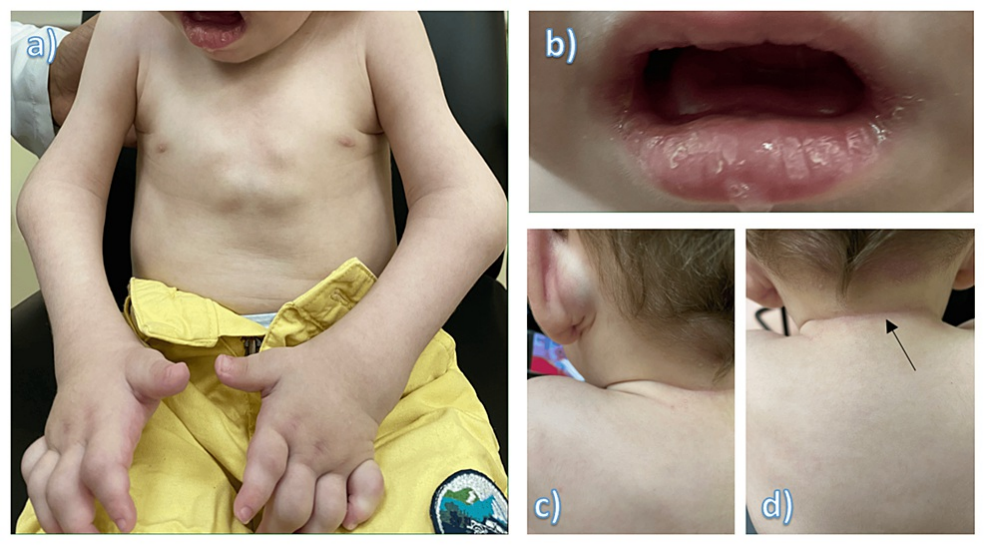
Physical examination findings a) chest nodules and bilateral contractures of elbow joints, b) gingival hypertrophy, c) left postauricular nodule, and d) skin hyperpigmentation (arrow)

The patient had been born at term via uncomplicated vaginal delivery. His birth weight had been 3.0 kg. Upon his first visit to our clinic at six months of age, he weighed 4.9 kg, which was well below the third percentile for his age. On physical examination, he had multiple skin lesions on the face, trunk, and extremities, ranging from flat, flesh-colored papules to larger nodules with a waxy appearance. The skin lesions were firm to palpation and showed no evidence of ulceration or discharge. The skin was also thickened, with hyperpigmentation over the joints. Another prominent feature was gingival hypertrophy. The patient had severe stiffness of both upper and lower limbs and joint contractures involving the elbows, knees, and ankles, with a limited range of motion (Figure 2).

The cardiovascular examination revealed the presence of a continuous murmur that was best heard in the left infraclavicular area. The initial laboratory workup was significant for low levels of serum albumin (2.1 g/dL). Other routine laboratory results, including complete blood count, electrolytes, renal function tests, and serum calcium, were within normal limits. MRI brain revealed symmetric periventricular hyperintensities. MRI spine showed soft tissue thickening and heterogeneity at the sacrococcygeal junction level extending distally. A patent foramen ovale with a left-to-right shunt was detected on echocardiography.

Based on the clinical presentation, the diagnosis of HFS was suspected. Accordingly, a skin biopsy was performed, which showed diffuse deposition of eosinophilic material in the dermis and subcutaneous tissues, consistent with hyaline fibromatosis. Genetic testing confirmed the diagnosis of HFS, with a homozygous mutation in the ANTXR2 gene.

The patient was managed with supportive care, including moisturizers and intralesional corticosteroids for the skin nodules, and physiotherapy for the joint contractures. Physiotherapy sessions were given at regular intervals with follow-up appointments at the pediatric outpatient clinic. A pediatric gastroenterologist and a nutritionist were also involved in the management of the patient’s faltering growth. The patient also received multiple albumin infusions to correct his low serum albumin levels. Gingival hypertrophy was managed with regular dental care. The patient was referred to a pediatric cardiologist for the management of the patent foramen ovale. The parents were referred to a genetic counselor who provided them with more information regarding the pattern of inheritance and the risk of having another child with HFS.

**Figure 3:**
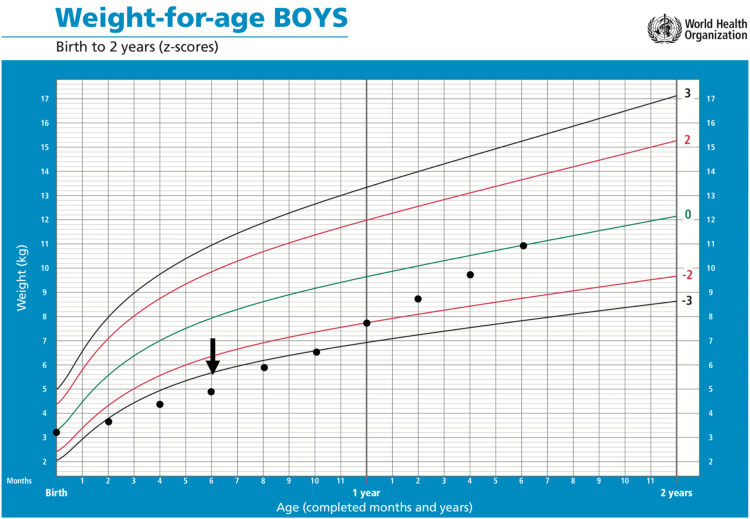
World Health Organization growth chart of a 1.5-year-old Bahraini male with hyaline fibromatosis syndrome The chart shows initial weight faltering followed by improvement in his weight after nutritional support starting at the age of six months (arrow) Source: WHO Child Growth Standards. https://www.who.int/tools/child-growth-standards/standards

At the follow-up at 1.5 years of age, the patient responded to the regular physiotherapy sessions and showed some improvement in joint mobility and range of motion. Multiple skin lesions regressed upon the administration of intralesional corticosteroid injections. After extensive nutritional support and multiple albumin infusions, the patient was able to gain weight, as presented in the World Health Organization growth chart below (Figure 3). 

The patient's gingival hypertrophy remained stable with regular dental care. His patent foramen ovale was managed conservatively and did not require intervention. A repeat Brain MRI showed no progression of the periventricular hyperintensities.

## Discussion

While the exact prevalence of HFS is unknown, it is estimated to be less than one in 1,000,000 individuals worldwide [4]. This is the second case of HFS reported in Bahrain, but the first to present with severe gastrointestinal manifestations [5]. The relatively higher prevalence of HFS in this region could be attributed to the higher rates of consanguineous marriages.

Previously, the syndrome was classified into two separate entities: “infantile type” and “juvenile type” [6]. Common manifestations between the two entities include the development of skin nodules and ulceration, joint contractures, bony deformities, and hyperpigmented skin over bony prominences [7]. The symptoms of infantile HFS, as in this case, usually manifest during early infancy. Severe cases may also present with faltering growth [8]. Faltering growth is thought to be caused by various factors. One of the primary causes is malabsorption, which in turn is due to the accumulation of hyaline material in the gastrointestinal tract. Hyaline material can lead to thickening and fibrosis of the intestinal walls, which can impair the absorption of nutrients from the diet. The accumulation of hyaline material in the liver can also lead to liver dysfunction, which can further contribute to malabsorption [9]. In addition to structural changes in the gastrointestinal tract, HFS can also lead to functional abnormalities that contribute to faltering growth. For example, infants with HFS often have feeding difficulties due to oral and pharyngeal muscle weakness and gastroesophageal reflux. Juvenile HFS tends to be milder than the infantile type and presents later in childhood [10].

The management of HFS is mainly supportive, and there is no cure for this disorder [7-10]. A multidisciplinary approach is required for the management of faltering growth to address the underlying causes of poor growth and development, as well as the associated medical complications. Our patient was referred to a pediatric gastroenterologist and nutritionist, both of whom provided him with extensive nutritional support whereby he was given elemental formula and multiple albumin infusions to correct his low albumin levels. A case of infantile HFS reported by Al Kaissi et al. was managed in a similar manner [11]. Nutritional assessment and intervention, including the use of specialized formulas or enteral feeding, may be necessary to ensure adequate caloric intake and promote growth. Physical therapy is also an important component. It can help improve muscle strength, range of motion, and coordination, which can enhance feeding ability and promote weight gain. Gastrointestinal procedures, such as nasogastric tube or gastrostomy tube feeding, may be required in cases with severe feeding difficulties [7,8,11].

Joint contractures are mainly managed with physiotherapy, with the aim of improving joint mobility and muscle strength [11]. In our case, the patient’s joint contractures were managed with regular physiotherapy sessions, which resulted in some improvement in his joint mobility. Several other studies, including one by Rangel Rivera et al., have reported the effectiveness of physiotherapy in promoting joint mobility and muscle strength [12-14]. However, the optimal type, frequency, and duration of physiotherapy interventions are not well established, and further research is needed to evaluate the effectiveness of different physiotherapy interventions in HFS. Some studies have reported the use of penicillamine to improve joint contractures. Nonetheless, data on its effectiveness remains limited [14]. In severe cases, surgical intervention may be necessary to correct bone deformities and improve joint mobility. Surgical options include joint release or replacement, tendon transfers, and skin grafting. Surgery is typically considered a last resort due to the potential risks and complications associated with these procedures.

Skin nodules were traditionally managed with surgical excision, although some studies have reported a recurrence of nodules post-surgical excision [13-15]. In our case, multiple skin nodules were treated with intralesional corticosteroid therapy, especially the ones next to the chest area which were difficult to excise surgically due to their location. Our patient responded well to the corticosteroid therapy, which resulted in a decrease in the size of nodules. Braizat et al. have reported the effectiveness of corticosteroids in the management of skin nodules in a case of juvenile HFS [15].

Other modalities used to treat HFS include regular dental care to manage gingival hypertrophy, as well as genetic counseling [16]. The stress and challenges associated with caring for an infant or young child with a complex medical condition can have a significant impact on the mental health and well-being of parents and caregivers [17]. Access to support groups, counseling, and respite care can help reduce caregiver burden and improve the overall quality of life for families affected by HFS.

## Conclusions

HFS is a rare genetic disorder characterized by multisystemic manifestations. While the clinical presentation and prognosis may vary, early multidisciplinary management is crucial to improve the quality of life of affected individuals since there is currently no cure for this condition. The treatment focuses on managing the symptoms through physiotherapy, analgesics, nutritional support, regular dental care, and psychosocial support. Severe bone deformities and joint contractures may require surgical management. Corticosteroid therapy may be effective for some skin nodules, while surgical excision may be necessary for others. Ultimately, further research is needed to identify more effective treatment options and to better understand the underlying mechanisms of the disease.
